# Iron status modulates cadmium tolerance: mechanistic insights from *MsbHLH60*-mediated regulation of the FIT/IRT1 module in alfalfa

**DOI:** 10.3389/fpls.2026.1821873

**Published:** 2026-05-08

**Authors:** Xin Liu, Menghan Chang, Wenzhe Du, Gemeng Yu, Yuzhe Yang, Liang Si, Changhong Guo, Yingdong Bi, Donglin Guo

**Affiliations:** 1 Heilongjiang Provincial Key Laboratory of Molecular Cell Genetics and Genetic Breeding, College of Life Science and Technology, Harbin Normal University, Harbin, China; 2 Institute of Crops Tillage and Cultivation, Heilongjiang Academy of Agricultural Sciences, Harbin, China

**Keywords:** alfalfa, bHLH transcription factors, cadmium stress, iron deficiency, *MsbHLH60*, transcriptional regulation

## Abstract

**Introduction:**

Basic helix-loop-helix (bHLH) transcription factors play pivotal roles in plant adaptation to metallic elements, yet the mechanisms by which leguminous bHLHs regulate iron (Fe) homeostasis and cadmium (Cd) stress adaptation remain inadequately understood.

**Methods:**

MsbHLH60 and its promoter was cloned from alfalfa. The expression of *MsbHLH60* under iron deficiency (–Fe), Cd, and Cd accompanied by Fe deficiency (Cd(–Fe)) was analyzed by qRT-PCR and GUS assay. MsbHLH60 was subcellular localized. Overexpressing *MsbHLH60* Arabidopsis was generated and subjected to phenotypic and physiological assays under –Fe, Cd and Cd(–Fe) stress. The interaction was studied through Y2H, Y1H and transient co-expression.

**Results:**

MsbHLH60 belongs to bHLH transcription factor family and was localized to nucleus and cytoplasm. *MsbHLH60* was strongly expressed in the young tissues of alfalfa, and was significantly upregulated in roots by –Fe or Cd stress, especially in the stele. However, *MsbHLH60* was not induced by Cd(–Fe). Overexpression of *MsbHLH60* enhanced Arabidopsis tolerance to –Fe and Cd stress. *MsbHLH60* mediated Cd tolerance was notably attenuated by –Fe. MsbHLH60 could interact with MsFIT and enhanced the activate effect on *MsIRT1* expression.

**Discussion:**

The results suggested that *MsbHLH60* might serve as an auxiliary regulator in the Fe regulation network and might orchestrate plant adaptive response to –Fe and Cd by modulating metal transport and distribution and enhancing antioxidant capacity. This study provides novel insights into the intersecting mechanisms of nutrient homeostasis and metal stress tolerance in plants, laying a theoretical foundation and genetic resources for crop improvement.

## 1 Introduction

Iron (Fe) is a crucial trace nutrient for plants, acting as a cofactor in various biological processes, including chlorophyll synthesis (Gao and Dubos, 2021). Iron deficiency disrupts the electron transport chain, leading to increased reactive oxygen species (ROS), reduced photosynthesis, leaf yellowing, and stunted growth (Sood, 2025; Zhang et al., 2025c). Non-Poaceae plants use Strategy I for Fe absorption: H^+^-ATPase acidifies the rhizosphere, Fe oxidoreductase converts Fe^3+^ to Fe^2+^, and IRT transporters move Fe^2+^ into root cells (Pu and Liang, 2023). Absorbed Fe is chelated by nicotinamide and transported by yellow stripe proteins for redistribution (Ibeas et al., 2019). Plants also adopt adaptive strategies to expand the area for Fe absorption by promoting auxin accumulation at the root tips and stimulating lateral root formation (Roman et al., 2023).

Cadmium (Cd) is a non-essential and highly toxic heavy metal that poses significant ecological risks to plant systems (Sterckeman and Thomine, 2020; Zhao et al., 2022). Cd exposure also triggers the overproduction of ROS, which impair membrane integrity, inhibit enzyme activities, and reduce photosynthetic efficiency (Li et al., 2023b; Liu et al., 2024a). In addition, Cd interferes with hormone signaling pathways, notably reducing root elongation and fresh biomass accumulation (Zhang et al., 2024b; Xia et al., 2025; Hu et al., 2025). Given that Cd is a non-essential metal to plants but possesses a highly similar Ion hydration radius with Fe, Mn and Zn, Cd evolutionarily does not have specific transporters for its uptake and translocation. Cd competes with essential micronutrients—particularly Fe, Zn, and Mn—for uptake via key transport proteins such as ZIP, IRT, and NRAMPs. This competitive inhibition disrupts plant mineral nutrition and leads to nutrient imbalance (Tan et al., 2020; Yu et al., 2024). To counteract Cd toxicity, plants activate defense mechanisms, including the upregulation of detoxification-related genes (Liu et al., 2017; Yu et al., 2024). Among these, antioxidant enzymes and detoxifying enzymes such as Glutathione S-Transferase (GST) play critical roles in scavenging Cd-induced ROS (Fang et al., 2020; Li et al., 2023b; Wang et al., 2023a). Furthermore, basic helix-loop-helix (bHLH) transcription factors such as FIT, AtbHLH38, AtbHLH39, bHLH104 and bHLH115 are increasingly recognized as regulators in plant responses to Cd stress (Wu et al., 2012; Zhang et al., 2015, 2024a; Yao et al., 2018). They function by optimizing Fe homeostasis, maintaining overall metal ion balance, activating detoxification and antioxidant pathways, and cross-regulating hormone signaling networks, thereby enhancing plant tolerance to Cd toxicity (Wu et al., 2012; Sabella et al., 2021; Zhang et al., 2024a; Zhang and Lu, 2024).

In recent decades, significant progress has been made in understanding the mechanism of Cd accumulation in plants. The antagonistic relationship between Fe and Cd helps to reduce Cd toxicity in plants and maintain mineral nutritional balance. Studies on model species Arabidopsis and rice have revealed the role of Fe transporters in Cd absorption and Cd sequestration in roots. In this way, a thorough understanding of the regulatory mechanism of the mutual interference between Fe and Cd in plants becomes particularly important, and this knowledge needs to be extended to other species (Prasad, 1995; Sterckeman and Thomine, 2020; Sun et al., 2025). In conditions where Fe is abundant, Fe^2+^ ions compete with Cd^2+^ ions for binding sites on transport proteins such as Iron-Regulated Transporter 1 (IRT1) and Natural Resistance-Associated Macrophage Protein (NRAMP), thereby diminishing the passive uptake of Cd^2+^. Additionally, Fe may mitigate Cd toxicity by enhancing antioxidant defenses and facilitating the compartmentalization of heavy metals into vacuoles (Wu et al., 2026). Conversely, in iron deficiency, transport proteins like IRT1 and NRAMP are upregulated, which not only ameliorates iron deficiency but also increases the uptake rate of Cd. This upregulation exacerbates oxidative stress, thereby increasing plant sensitivity to Cd (Clemens and Ma, 2016; Zhang et al., 2020; Liu et al., 2022b).

Throughout evolution, plants have developed a sophisticated molecular network centered on basic Helix-Loop-Helix (bHLH) transcription factors to regulate iron uptake and maintain iron homeostasis. In model organisms such as Arabidopsis, functional analyses of *FIT* and *bHLH38*/*39*/*100*/*101* have elucidated the fundamental mechanisms by which plants respond to iron deficiency. Upon sensing signals of iron deprivation, these transcription factors form specific heterodimers that directly activate the expression of various downstream genes, including iron chelate reductase (e.g., *FRO2*) and iron transporter (e.g., *IRT1*) genes, thereby facilitating the efficient absorption of iron by the root system (Gao et al., 2019). In Gramineous plants, the mechanisms of iron absorption and transport differ from those observed in Arabidopsis, which utilizes phytosiderophore. Nevertheless, a regulatory network centered around the bHLH transcription factor is also present. For instance, *IRO2* and *OsbHLH133* enhance iron utilization by activating phytosiderophore synthesis genes and modulating iron distribution between roots and shoots (Lei et al., 2020; Li et al., 2022b). *OsIRO3* acts as a negative regulator of *OsIRO2*, thereby preventing excessive iron accumulation (Li et al., 2022a). Additionally, bHLH104 interacts with the auxin signaling protein ILR3 to maintain a balance between iron uptake and toxicity (Zhang et al., 2015; Pu and Liang, 2023). The bHLH transcription factors are also subject to post-translational regulation and epigenetic modifications, which play a crucial role in maintaining iron homeostasis in response to fluctuating iron availability (Gao et al., 2020; Shee et al., 2022). In response to the challenge that iron transport proteins also facilitate Cd uptake, plants have evolved specific coordination strategies (Clemens and Ma, 2016; Huang et al., 2024). FIT and Ib bHLH proteins (e.g., bHLH38/39) integrate iron homeostasis signals and indirectly modulate Cd toxicity. The expression of *FIT* and Ib bHLH transcription factor genes is upregulated upon exposure to Cd (Wang et al., 2023b). Furthermore, *bHLH104* enhances Cd tolerance in plants by upregulating *HMA3* and *NAS4* expression, thereby increasing root Cd sequestration and reducing its accumulation in shoots (Zhang et al., 2015; Yao et al., 2018). Certain bHLH transcription factors involved in iron regulation also modulate antioxidant and detoxification pathways, playing a crucial role in the plant response to Cd stress.

Transcriptional analyses have identified key genes differently expressed under Cd stress, including five bHLH transcription factors regulated by iron deficiency, as well as FRO2 and IRT1 (Zhang et al., 2024a). A bHLH transcription factor, *GmORG3* mediates Cd tolerance by regulating iron homeostasis, restricting root to shoot Cd translocation and alleviating Cd induced physiological damage (Xu et al., 2017). Recent studies have increasingly elucidated that certain bHLH members can directly or indirectly perceive heavy metal stress and mitigate toxicity by regulating the expression of detoxification-related genes, such as plant chelate synthase (PCS), heavy metal-associated isoprene plant protein (HIPP), and GST (Vitelli et al., 2024; Xia et al., 2025; Hu et al., 2025). The convergence and overlap of this regulation strongly indicate the presence of a fundamental regulatory module in plants, predominantly governed by the bHLH transcription factor, which links nutrient perception with stress adaptation. Consequently, investigating the mechanisms by which specific bHLH transcription factors detect and integrate iron nutritional status with heavy metal stress signals—thereby achieving a precise equilibrium between “source enhancement” (facilitating iron uptake) and “throttling/detoxification” (mitigating Cd toxicity) at the molecular level—has emerged as a forefront area of research in plant nutritional genetics and environmental stress biology. The “FIT-IRT1” regulatory pathway demonstrates significant adaptability across species, from Arabidopsis, a model dicotyledon, to members of the gramineae family, representing monocotyledons. Although there is evidence suggesting a close relationship among the bHLH transcription factor, iron homeostasis and Cd detoxification in Arabidopsis and cereal crops, the understanding of the detailed molecular regulation framework in Fe-Cd signal crosstalk remains limited, especially in leguminous plants such as alfalfa.

Alfalfa is a perennial tetraploid leguminous plant with a large genome, making its molecular research rather challenging. However, alfalfa has a high tolerance to heavy metals and a high efficiency in heavy metal absorption, and thus holds great potential in the phytoremediation of heavy metal-contaminated soil. Therefore, alfalfa is still regarded as an excellent material for studying plant metal stress, nutritional physiology and molecular regulation (Gao et al., 2023; Liu et al., 2024b; Zhang and Wang, 2025; Zhang et al., 2025b). Our previous study confirmed that overexpression of *MsbHLH115* enhanced Cd tolerance of Arabidopsis (Zhang et al., 2024a). However, there is insufficient understanding of the bHLH members function in regulating Fe-Cd tolerance in alfalfa. The analysis of the regulatory role and adaptation mechanism of bHLHs under combined Fe deficiency and Cd stress is still limited. Through transcriptomic analysis, we have identified an iron deficiency response gene, *MsbHLH60*. This study focuses on the regulatory role of *MsbHLH60* in iron homeostasis and Cd tolerance, as well as its interactions with other bHLH proteins and downstream target genes, aiming to enrich the bHLH-mediated Fe-Cd regulatory network in leguminous plants and provide candidate genes for improving Fe-utilization and Cd-phytoremediation in plants.

## 2 Materials and methods

### 2.1 Plant materials and growth conditions

Seeds of alfalfa (*Medicago sativa* cv. Longmu803) were kindly provided by the Animal Husbandry Research Institute of Heilongjiang Academy of Agricultural Sciences. Seeds of soybean (*Glycine max*. Heinong51) were kindly provided by the Soybean Research Laboratory of the Cultivation and Tillage Institute of the Heilongjiang Academy of Agricultural Sciences. Seeds of Arabidopsis (Col-0) and *Nicotiana benthamiana* were preserved in our laboratory.

Alfalfa seeds were surface-sterilized with 70% ethanol and germinated on moistened filter paper at 22 °C in the dark. Seedlings at the 2~4-leaf stage were transferred to 1/2 Hoagland nutrient solution, which was renewed every 3 days. Roots and leaves were separately harvested and stored for quantitative analysis.

Soybean seeds were sown in a mixed substrate (commercial nutrient soil: vermiculite = 1:1 by volume) and irrigated every 3 days with 1/2 Hoagland nutrient solution. Plants were grown at 22 °C under a 16 h light/8 h dark photoperiod. Soybean seedlings with unexpanded cotyledons were selected for subsequent infection assays.

Wild-type (WT) Arabidopsis were sown in a mixed substrate (commercial nutrient soil: vermiculite = 1:1 by volume) and irrigated every 7 days with 1/2 Hoagland nutrient solution. Plants were cultivated under controlled conditions at 22 °C under a 16 h light/8 h dark photoperiod. For subsequent infection assays, plants were used when the primary inflorescence had elongated and produced a few open flowers, while still bearing abundant unopened floral buds.

### 2.2 Cloning of the *MsbHLH60* and its promoter

Total RNA was extracted from alfalfa plants hydroponically cultured for 2 weeks using the Total RNA Kit II (Omega BioTek, Norcross, GA, USA) and reverse transcribed into cDNA using ReverTra Ace™ qPCR RT Master Mix (TOYOBO, Osaka, Japan). The coding sequence of *MsbHLH60* was amplified from cDNA. The total DNA of alfalfa was extracted using the E.Z.N.A.^®^ SP Plant DNA Kit (OMEGA, New York, NY, USA). The promoter of *MsbHLH60* (*MsbHLH60*pro) was amplified from the genomic DNA. The primers are shown in **Table 1**. After purification, PCR products were sequenced (Sangon Biotechnology Co., Ltd., Shanghai, China).

**Table 1:** Analysis of cis-regulatory elements in the *MsbHLH60* promoter.

Category	Name	Number
Plant growth and development	AAGAA-motif	2
	ACE	1
	Box 4	2
	chs-CMA1a	1
	G-box	2
	GT1-motif	1
Abiotic and biotic stress responses	MBS	1
	MRE	1
	TC-rich repeats	2
	STRE	1
	E-box	4
Plant hormone response	ABRE	2
	MYC	4
	TGACG-motif	1
	CGTCA-motif	1

### 2.3 Bioinformatic analysis of the *MsbHLH60* gene and its promoter

The genome sequence, CDS, protein sequence, and gff file of the *M. sativa* were obtained using the MODMS platform (https://modms.lzu.edu.cn). The hidden Markov model (HMM) file (PF0010) for the bHLH structure was downloaded from the InterPro database (https://www.ebi.ac.uk/interpro/) and subsequently used for screening with HMMER3.4 software to identify candidate sequences containing the bHLH_AtbHLH_like domain. Redundant sequences were removed using NCBI-CDD (https://www.ncbi.nlm.nih.gov/Structure/cdd/wrpsb.cgi) and SMART (https://smart.embl.de/). The physicochemical properties of the bHLH protein were predicted using the ProtParam tool on the ExPASy website (http://web.expasy.org/protparam/).

A phylogenetic tree was constructed using the ClustalW tool in MEGA, and the visualization was enhanced in Adobe Illustrator. Conserved motifs in the protein were analyzed with the MEME online software (http://meme-suite.org/index.html), and gene structure along with conserved domains were visualized using TBtools (Chen et al., 2023). Additionally, inter- and intra-species collinearity analyses were performed using TBtools, and the chromosomal locations of each gene were determined. The identified bHLH proteins were sorted by chromosomal location, and the corresponding bHLH genes were named accordingly. Msa0900200 (the gene exhibiting an intraspecific duplication relationship with MsbHLH60) and MsbHLH61 (the phylogenetically closest homolog of MsbHLH60) were identified through intraspecific synteny analysis and phylogenetic clustering, respectively.

Homologous sequences from other species—including *M. sativa*, *M. truncatula*, and Arabidopsis were analyzed by NCBI blast and MODMS blast. Multiple sequence alignment of these sequences was conducted using DNAMAN. The nuclear localization signal (NLS) of MsbHLH60 protein was predicted using cNLS Mapper (https://nls-mapper.iab.keio.ac.jp/cgi-bin/NLS_Mapper_form.cgi). The SUMOylation sites of the MsbHLH60 were predicted using the GPS-SUMO 2.0 online tool (http://sumo.biocuckoo.cn/advanced.php). The phosphorylation sites of the MsbHLH60 were predicted using the NetPhos 3.1 server (http://www.cbs.dtu.dk/services/NetPhos/). The cis-regulatory elements within *MsbHLH60* promoter were predicted using the PlantCARE website (http://bioinformatics.psb.ugent.be/webtools/plantcare/html/).

### 2.4 Subcellular localization of MsbHLH60 protein

The *MsbHLH60* without the stop codon was ligated into the pBI121-GFP vector using the restriction enzymes *Xba* I and *Sma* I. The pBI121-*MsbHLH60*-GFP plasmid was transformed into *A. tumefaciens* GV3101 via the freeze-thaw method, and positive clones were screened by colony PCR. A single colony of GV3101 harboring pBI121-*MsbHLH60*-GFP was picked, activated in liquid medium until the OD_600_ reached 1.0, and then centrifuged at 5000 rpm for 5 min. The pellet was re-suspended to an OD_600_ of approximately 0.8, kept in the dark for 3 h, and then injected into leaves of *N. benthamiana* at 4~6 leaf stage. After 48 h, observations were performed using a Leica TCS SP8 confocal microscope with an excitation wavelength of 488 nm and 561 nm.

### 2.5 Plant transformation

The *MsbHLH60* CDS was inserted into the pBI121 vector via the *Xba* I and *Sma* I sites. The pMD18T-*MsbHLH60*pro fragment was inserted into the pBI121::GUS vector via the *Bgl* II and *Xba* I sites. The plasmids pBI121-*MsbHLH60* and pBI121-*MsbHLH60*pro::GUS were transformed into GV3101 via the freeze-thaw method, and *MsbHLH60* transgenic Arabidopsis plants were obtained using the floral dip method. The *MsbHLH60*-transformed Arabidopsis was detected by PCR and qRT-PCR until T_3_ lines were obtained. The primer used was shown in **Table 1**.

### 2.6 Soybean hairy root chimeras induction

Before the soybean cotyledons were fully expanded, the cotyledonary nodes were infected via needle injection with *A. tumefaciens* K599 harboring pBI121-*MsbHLH60*pro::GUS (OD_600_≈ 0.8) to obtain transgenic soybean hairy root chimeras. The chimeras were divided into four groups and treated with: (1) Normal condition (N, 100 µM Fe-EDTA); (2) Iron deficiency (−Fe, 0 µM Fe-EDTA); (3) Cd stress (Cd, 100 µM Fe-EDTA and 75 µM CdCl_2_); and (4) Cd stress with iron deficiency (Cd(−Fe), 0 µM Fe-EDTA and 75 µM CdCl_2_) for 1 day, respectively. The experiment was independently repeated three times.

### 2.7 GUS staining

According to the instructions of the GUS staining kit (SL7160), X-Gluc powder was diluted and mixed with the buffer solution to prepare the GUS staining solution. Plant materials were completely immersed in the staining solution and incubated at 28 °C for 24 h, then placed in 95% alcohol until the green color was disappears. Observations and photography were performed using a microscope (NOVEL NSZ-608T).

### 2.8 Quantitative real-time PCR analysis

For Quantitative real-time PCR (qRT-PCR of *MsbHLH60*, two-week-old hydroponically grown alfalfa seedlings were divided into four groups and treated with: (1) N, 100 µM Fe-EDTA; (2) −Fe, 0 µM Fe-EDTA; (3) Cd, 100 µM Fe-EDTA and 90 µM CdCl_2_; (4) Cd(−Fe), 0 µM Fe-EDTA and 90 µM CdCl_2_, respectively. Leaves and roots were collected at 0, 6, 12, 24, 48, and 72 hours after treatment. The expression of *MsbHLH60*, *AtFIT*, and *AtIRT1* in rosette leaves of two-week-old *MsbHLH60*OE and WT plants were analyzed by qRT-PCR. The qRT-PCR was performed using a Bio-Rad CFX 96 detection system (Bio-Rad, Hercules, CA, USA) and Top Green qPCR Super Mix (Transgenic Biotechnology Company, Beijing, China). Gene expression was quantified using the 2^–ΔΔCT^ method with *MsActin11* as the internal reference gene. Primers were listed in **Table 1**. The experiment was independently repeated three times.

### 2.9 Physiological index measurement

T_3_ generation seeds of *MsbHLH60*OE and WT were sterilized with 10% sodium hypochlorite and cultured until the 2**~**4 leaf stage. These selected seedlings were transferred to MS medium supplemented with different stress conditions: (1)N, 100 µM Fe-EDTA; (2) −Fe, 0 µM Fe-EDTA; (3) Cd, 100 µM Fe-EDTA and 200 µM CdCl_2_; (4) Cd(−Fe), 0 µM Fe-EDTA and 200 µM CdCl_2,_ respectively. The concentration of CdCl_2_ was set based on reference (Zhang et al., 2024a) and our preliminary experiments. In the preliminary tests, 90 µM CdCl_2_ was used but did not induce an obvious phenotype; therefore, the concentration was gradually increased to 200 µM for the final treatments.

Under each stress condition, three biological replicates were set up for each line (WT, OE1, OE2, OE3). In each biological replicate, three randomly selected seedlings of uniform growth were used for the measurement. Phenotype of the plants were observed, and physiological and biochemical indices of rosette leaves were measured.

Total chlorophyll content was measured as described by Yavari et al (Yavari et al., 2021). The DAB staining solution was prepared by diluting 50 mM Tris-HCl to 1 mg/mL. The NBT staining solution was prepared by diluting 50 mM PBS to 0.5 mg/mL (pH 7.8) and used immediately (Soltabayeva and Sagi, 2024). The staining solution was stored in the dark at 28 °C for 6 hours, and then decolorized in a 70 °C water bath using 90% ethanol. The hydrogen peroxide (H_2_O_2_) content was measured according to the method described by Veljovic et al., and the superoxide anion (O_2_^-^) content was measured according to Nakajima et al (Veljovic Jovanovic et al., 2002; Nakajima et al., 2009). MDA content was determined using the method of Dhindsa (Dhindsa and Matowe, 1981). The GST activity was measured using the GST Assay Kit (M0305B, Mengxi Bio, Baoding, China). The activities of catalase (CAT) and superoxide dismutase (SOD, WST-1 method) were measured using their respective assay kits (CAT: BC0205; SOD: BC5165, both from Solarbio, Beijing, China).

### 2.10 Yeast two-hybrid and yeast one-hybrid

To screen for the interactions between MsbHLH60 and MsFIT protein, *MsbHLH60* was cloned into the pGBKT7 vector, and the pGBKT7-*MsbHLH60* vector was constructed. pGADT7-*MsFIT* used in this study was preserved in our laboratory. The combination of BD-*MsbHLH60* with AD-*MsFIT* served as the experimental group. AD-p53+BD-p53 were positive controls. AD+BD and AD+BD-*MsbHLH60* were negative controls. These vector combinations were co-transformed into the yeast AH109. The transformed yeast cells were cultured on SD/-Trp/-Leu and SD/-Leu/-Trp/-His/50 mM 3-Amino-1,2,4-triazole (3-AT) media. The protein interaction was verified by observing yeast growth.

To screen for the interactions between MsbHLH60 and *MsIRT1*pro, *MsbHLH60* was cloned into the pGADT7 vector, and the recombinant vector pGADT7-*MsbHLH60* was constructed. pHIS2-*MsIRT1*pro used in this study was preserved in our laboratory. AD-*MsbHLH60* + pHIS2-*MsIRT1*pro and AD-*MsFIT* + pHIS2-MsIRT1pro were served as the experimental groups. AD-p53+pHIS2-p53 were served as the positive control. AD+pHIS2-*MsIRT1*pro were served as the negative control. These vector combinations were co-transformed into the yeast Y187promoter. The transformed yeast cells were cultured on SD/-Trp/-Leu and SD/-Leu/-Trp/-His media. The protein and promoter interaction was verified by observing yeast growth.

### 2.11 Transient transformation of *N.benthamiana*

*A.tumefaciens* strain GV3101 harboring pBI121-*MsbHLH60*, pBI121-*MsFIT*, or pBI121-*MsIRT1*pro::GUS was cultured on solid LB medium with kanamycin at 28 °C for 48 hours. A single colony was picked and inoculated into 5 mL liquid LB medium containing kanamycin and rifampicin, followed by shaking incubation (200 rpm) at 28 °C for 24 hours. A 50 μL aliquot of the activated bacterial suspension was transferred to 50 mL liquid LB medium and cultured until the OD_600_ reached 1.0. Bacterial cells were harvested by centrifugation at 8000 rpm for 5 minutes, then resuspended in infiltration buffer (10 mM MgCl_2_, 10 mM MES, 200 μM acetosyringone, pH 5.6) to adjust the OD_600_ to 1.0. The resuspended cultures were incubated in the dark at room temperature for 3 hours. For the experimental groups, bacterial suspensions were mixed in equal volumes as follows: (1) *MsbHLH60*+*MsIRT1*pro::GUS; (2) *MsIRT1*pro::GUS; (3) *MsFIT+MsIRT1*pro*::GUS*; and (4) *MsbHLH60*+*MsFIT*+*MsIRT1*pro::GUS. The mixed bacterial suspensions were infiltrated into leaves of 4–6 leaf stage *N.benthamiana*. After infiltration, plants were incubated in the dark for 1 day, and then maintained under normal light for 2 days. Leaf samples were immersed in X-Gluc staining solution and incubated at 37 °C for 24 hours. Chlorophyll was removed by decolorization with 95% ethanol, and staining patterns were observed and documented using a stereomicroscope (NSZ-608T).

### 2.12 Statistical analysis

All data presented are the mean values ± SD. Each experiment included at least three independent biological replicates. Statistical analysis was performed using SPSS 19.0, and graphs were generated using GraphPad Prism. A Student’s t-test was used to compare transgenic plants and WT under the same treatment, and a one-way analysis of variance (ANOVA) followed by Duncan’s multiple range test was used to compare the control and treatment groups.

## 3 Results

### 3.1 Identification and bioinformatics analysis of *MsbHLH60*

**Figure 1:**
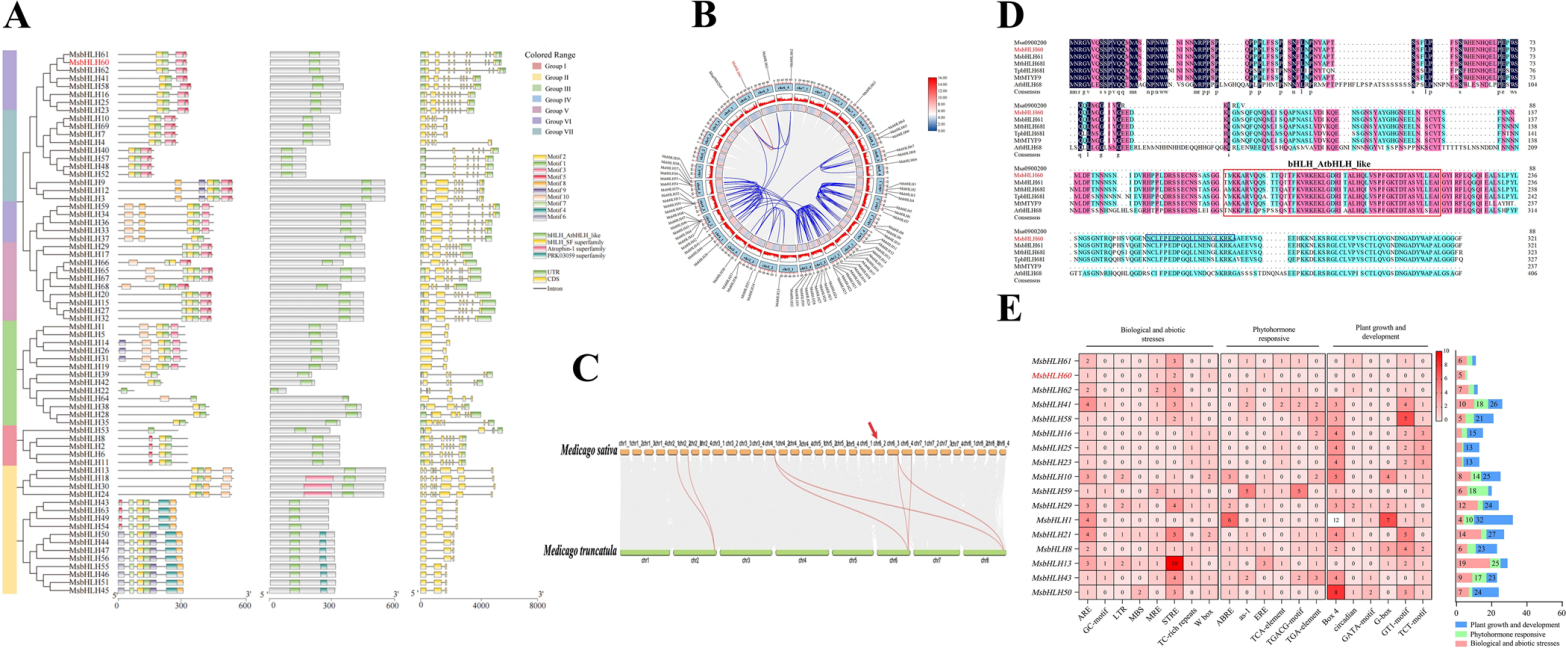
Identification and bioinformatics analysis of *MsbHLH60*. **(A)** Phylogenetic analysis, CDD, and motif analysis of 69 proteins in *Medicago sativa*. **(B)** Duplication relationships of bHLH genes in *Medicago sativa*. **(C)** Collinear relationships of selected bHLH gene family members between *Medicago sativa* and *Medicago truncatula*. Indicate MsbHLH60 with a red arrow. **(D)** Multiple sequence alignment of MsbHLH60 with its homologs. Seven homologs of MsbHLH60 were identified from *Medicago sativa*, *Medicago truncatula*, and *Arabidopsis thaliana* by NCBI BLAST and MODMS BLAST, including Msa0900200, MsbHLH60 (Msa0926800), MsbHLH61 (Msa0965520), MtbHLH68l (XP_013453115), TpbHLH68l (XP_045822568.1), MtMTYF9 (ACJ84970.1), and AtbHLH68 (NP_001328529.1). Indicate the bHLH_AtbHLH-like domain with the red box and the Nuclear Localization Signal (NLS) sequence in the blue box. **(E)** A total of 17 promoters were analyzed. The number of each cis-element is marked at the bottom, and the quantity corresponds from red to white. All the cis-elements are divided into three groups: abiotic and biotic stresses, phytohormone-responsive, and plant growth and development, respectively. The number of cis-elements in three groups is shown in the boxes on the right, with the length of each box corresponding to the number of cis-elements.

We obtained a novel bHLH TF CDS, which was named as *MsbHLH60* according to its chromosome location, from alfalfa (*M. sativa*) (**Supplementary Figures S1, S2**). *MsbHLH60* contained 981 bp, encoding a protein composed of 321 amino acid residues. The theoretical isoelectric point of MsbHLH60 is 5.72 and the relative molecular mass is 35.27 kDa (**Supplementary Table 2**). In the phylogenetic tree, the 68 MsbHLH proteins with bHLH_AtbHLH-like domain were divided into eight groups. Phylogenetic analysis revealed that MsbHLH60 and the other 7 MsbHLHs clustered together into one clade (**Figure 1A**). These proteins shared a similar motif distribution pattern, all containing Motif 1, Motif 2, Motif 3, and Motif 5. Gene structure analysis revealed that MsbHLH60 harbored 8 introns, a pattern consistent with MsbHLH61 (**Figure 1A**).

Collinearity analysis identified a gene duplication relationship between MsbHLH60 (located on chromosome chr6_2) and Msa0900200 (located on chromosome chr6_1) in *M. sativa* (**Figure 1B**). There was no syntenic relationship between MsbHLH60 and its homologs in *M. truncatula*. In contrast, Msa0900200 displayed synteny with MtbHLH68-like (Medtr8g103065.1) from *M. truncatula* (**Figure 1C**, **Supplementary Table 3**). The multiple sequence alignment showed that MsbHLH60 and its 5 homologous proteins contained the same bHLH_AtbHLH_like domain (**Figure 1D**), indicating that MsbHLH60 is a conserved bHLH_AtbHLH-like with an intact functional domain. Protein structure analysis also indicated that a nuclear localization signal (NLS) with the sequence “ENGLKVSQEEPKKDLK”, a serine phosphorylation site, and a ubiquitination site located at 253–274 aa of MsbHLH60 (**Figure 1D**; **Supplementary Figure S3**; **Supplementary Table 4**).

Promoter element analysis of *MsbHLH60* and its 16 homologs revealed that the STRE element was the most abundant in the categories of abiotic and biotic stress responses, the ABRE and TGA-element were the most frequent in the plant hormone response category, and the Box 4 element was the most numerous in the plant growth and development category (**Figure 1E**). Although there were only 6 cis-elements promoter in *MsbHLH6*0pro predicted by bioinformatics analysis using alfalfa genomic data, more stress response elements were discovered in cloned *MsbHLH6*0pro, in the category of plant growth and development, abiotic and biotic stress responses and plant hormone response (**Table 1**).

### 3.2 Subcellular localization of MsbHLH60

**Figure 2:**
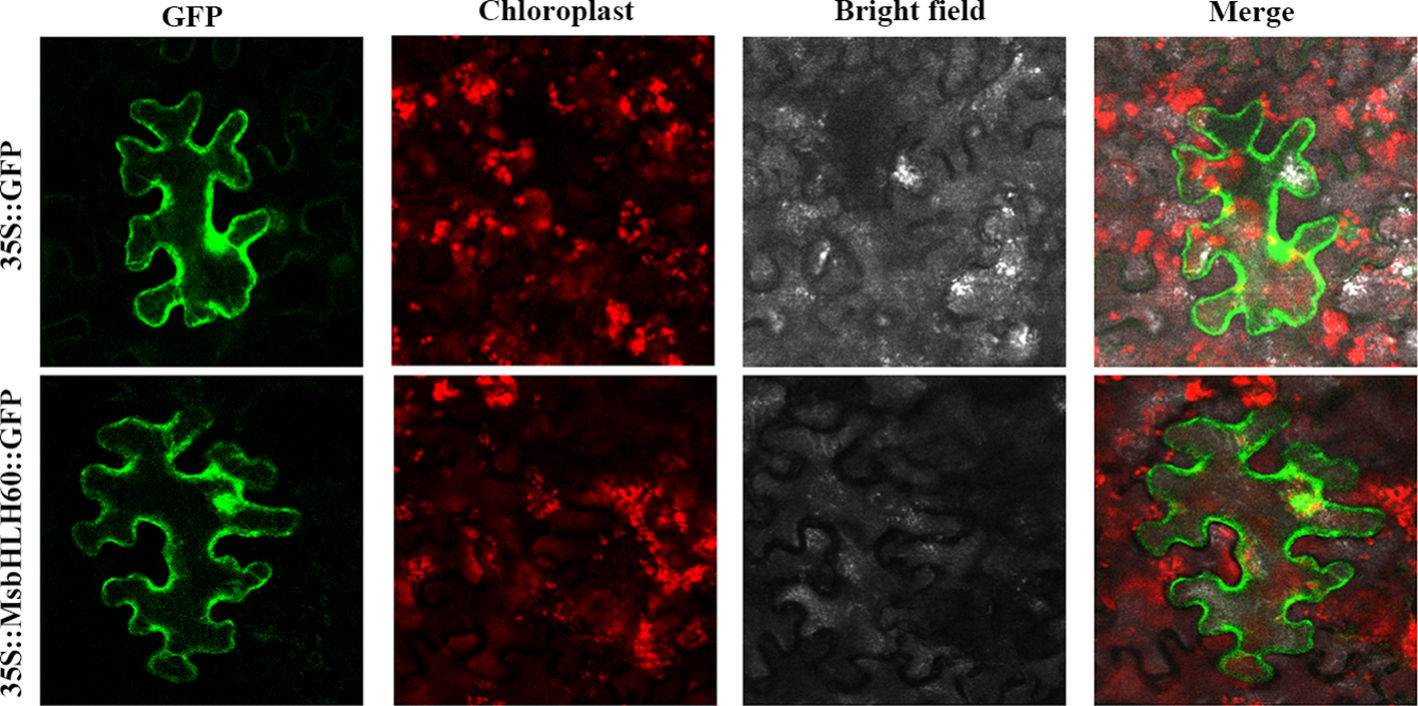
Subcellular localization of MsbHLH60. The subcellular localization of 35S::*MsbHLH60*-GFP and the control 35S::GFP was examined in transiently transformed Nicotiana benthamiana leaves using confocal laser scanning microscopy (Leica TCS SP8). GFP fluorescence (green) was excited at 488 nm, and chloroplast autofluorescence (red) was excited at 561 nm. Scale bars = 50 µm.

The pBI121-*MsbHLH60*-GFP expression vector was successfully constructed and transformed into *N. benthamiana* (**Supplementary Figure S4**). Microscopic observation revealed that the green fluorescence signal of Free-GFP was diffusely distributed throughout the entire cell of tobacco leaves, while the green fluorescence signal of MsbHLH60-GFP fusion protein was distributed in the nucleus and cytoplasm of tobacco leaves. The results indicated that MsbHLH60 was located in the nucleus and cytoplasm (**Figure 2A**).

### 3.3 Expression characteristics of *MsbHLH60* in response to iron deficiency

**Figure 3:**
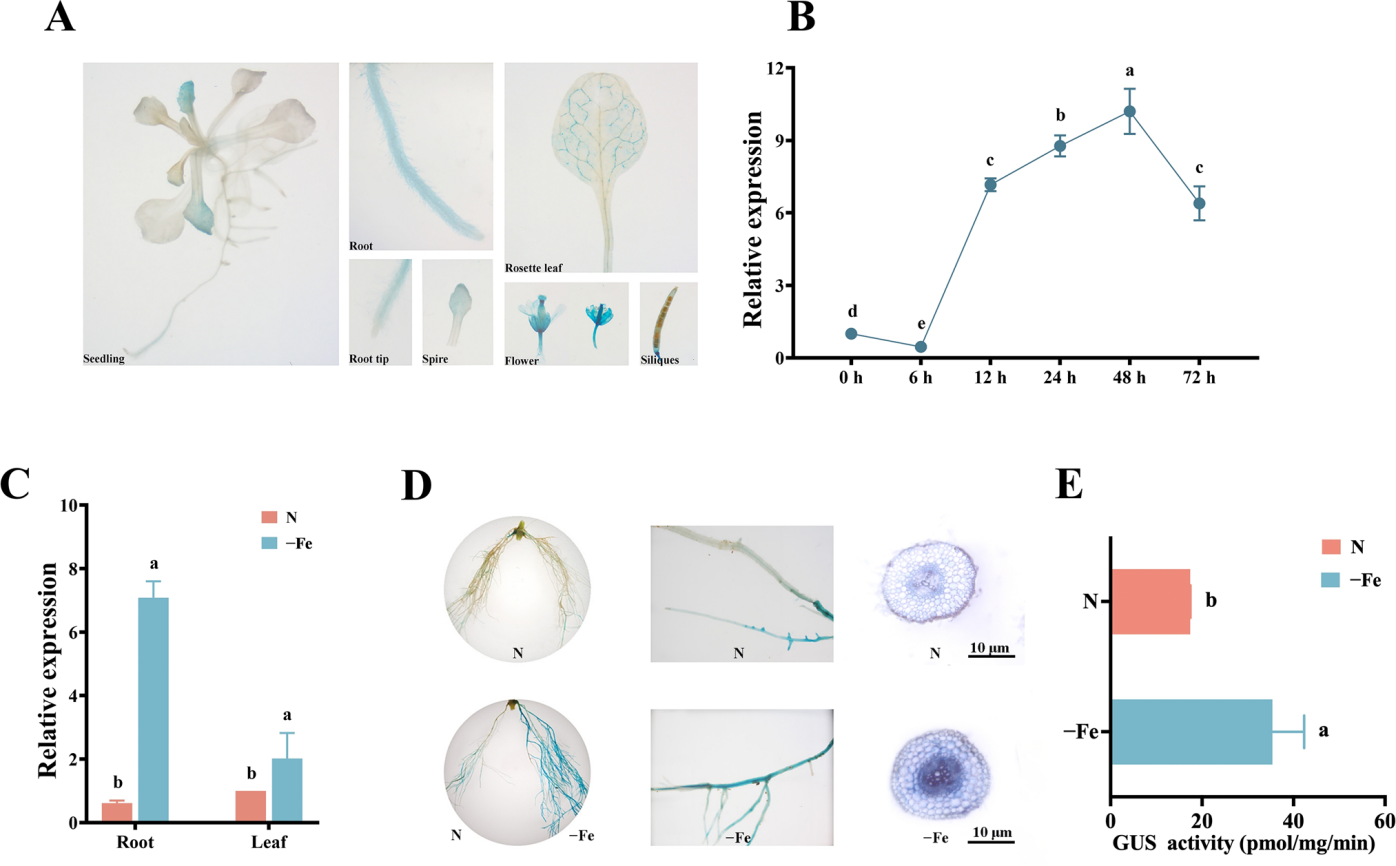
Expression characteristics of *MsbHLH60* in response to iron deficiency. **(A)** Representative images of GUS staining showing MsbHLH60 expression in different tissues of *Arabidopsis thaliana*: root, rosette leaf, root tip, inflorescence, flower, and silique. Scale bars=1 cm. **(B)** The relative expression of *MsbHLH60* in alfalfa seedlings under 0 µM Fe-EDTA stress (−Fe) for 0, 6, 12, 24, 48, and 72 (h) **(C)** The relative expression of *MsbHLH60* in the root and leaf of alfalfa under −Fe and non-treatment (N) for 24 (h) Statistical analysis was performed using SPSS 19.0, and graphs were generated using GraphPad Prism. A Student's t-test was used to compare transgenic plants and WT under the same treatment, and a one-way analysis of variance (ANOVA) followed by Duncan's multiple range test was used to compare the control and treatment groups. Different letters indicate a significant difference (*p* < 0.05). **(D)** GUS staining of soybean hairy root chimeras transformed with pBI121-*MsbHLH60*pro::GUS under –Fe and N for 24 (h) From left to right: whole-root morphology, local root segment details, and transverse root section. Scale bars = 10 µm. **(E)** GUS activity measurement. Statistical analysis was performed using SPSS 19.0, and graphs were generated using GraphPad Prism. A Student's t-test was used to compare transgenic plants and WT under the same treatment, and a one-way analysis of variance (ANOVA) followed by Duncan's multiple range test was used to compare the control and treatment groups. Different letters indicate a significant difference (*p* < 0.05).

To reveal the expression pattern of *MsbHLH60*, we performed GUS staining assay. The pBI121-*MsbHLH60*pro::GUS expression vector was successfully constructed (**Supplementary Figure S5**). In the transgenic Arabidopsis, strong blue staining was detected in the young roots, young leaves, calyces, and styles, while weak blue staining was detected in the root tips, rosette leaves, and siliques (**Figure 3A**). Further, the expression of *MsbHLH60* under–Fe was observed. Compared with untreated (0 h), the expression of *MsbHLH60* in alfalfa was significantly decreased at 6 h, while significantly increased at 12 h, 24 h, 48h, and 72 h (*p* < 0.05) (**Figure 3B**). *MsbHLH60* expression continued to rise from 6 h to 48 h by –Fe and then declined at 72 h. At 24 h of –Fe, the expression of *MsbHLH60* was significantly upregulated in both roots and leaves of alfalfa (*p* < 0.05), with a higher degree of induction in roots than in leaves (**Figure 3C**).

The pBI121-*MsbHLH60*pro::GUS was genetically transformed into soybean and generated hairy root chimeras. GUS staining revealed that under normal conditions (N), only a small portion of the *MsbHLH60*pro-transgenic soybean hairy root was stained blue. Compared with N, under −Fe, the GUS staining of the main roots and lateral roots was significantly deepened, accompanied by a significant increase in GUS enzyme activity. The GUS staining was significantly deepened at the stele of the cross-cut roots (**Figures 3D, E**).

### 3.4 The effect of *MsbHLH60* on the growth of Arabidopsis under iron deficiency

The pBI121-*MsbHLH60* recombinant vector was constructed (**Supplementary Figure S6**). Using Agrobacterium-mediated transformation, *MsbHLH60* transgenic Arabidopsis (*MsbHLH60*OE) were obtained. The T_3_ generation was obtained and detected by PCR (**Supplementary Figure S7**). The expression levels of *MsbHLH60* in three independent lines (OE1, OE2, and OE3) were significantly (*p* < 0.05) higher than those in WT by qRT-PCR detection.

**Figure 4:**
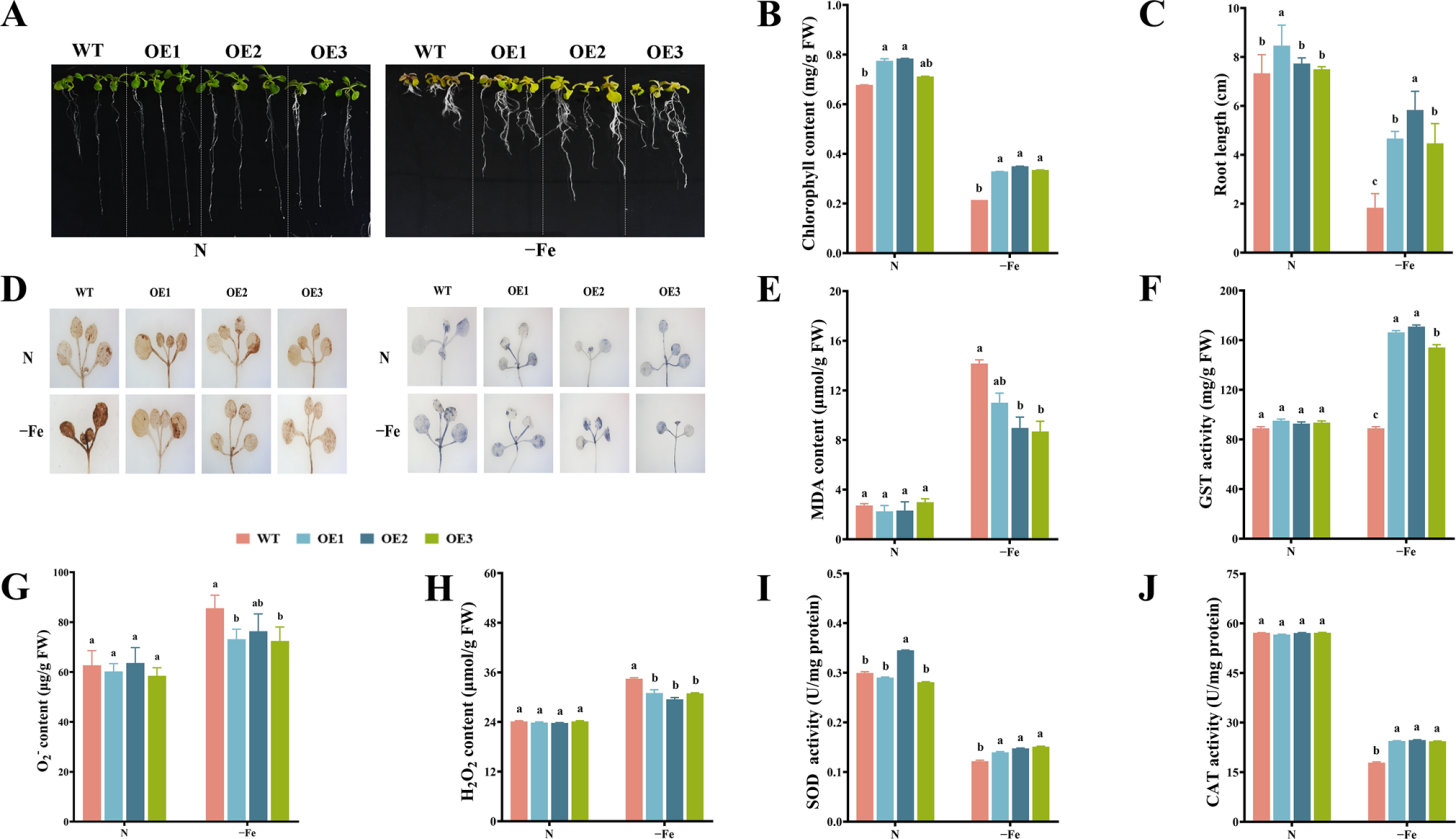
The effect of *MsbHLH60* on the growth of Arabidopsis under iron deficiency. **(A)** Phenotypes of *MsbHLH60*OE and WT Arabidopsis seedlings under −Fe and (N) OE1, OE2, and OE3: T_3_ generation lines of the *MsbHLH60* transgenic Arabidopsis. **(B)** Chlorophyll content. **(C)** Root length. **(D)** DAB and NBT staining. **(E)** MDA content. **(F)** GST activity. **(G)** O_2_^−^ content. **(H)** H_2_O_2_ content. **(I)** SOD activity. **(J)** CAT activity. Statistical analysis was performed using SPSS 19.0, and graphs were generated using GraphPad Prism. A Student's t-test was used to compare transgenic plants and WT under the same treatment, and a one-way analysis of variance (ANOVA) followed by Duncan's multiple range test was used to compare the control and treatment groups. Different letters indicate a significant difference (*p* < 0.05).

Under N, *MsbHLH60*OE plants showed better growth state than WT. The leaf color of *MsbHLH60*OE and WT turned yellow by −Fe, with greener leaves in *MsbHLH60*OE than in WT (**Figure 4A**). Under N, the chlorophyll content of *MsbHLH60*OE plants was significantly higher than that of WT (*p* < 0.05). −Fe led to a decrease in chlorophyll content of all plants, and the chlorophyll content of *MsbHLH60*OE was still significantly higher than WT (*p* < 0.05) (**Figure 4B**). Under N, the root length of *MsbHLH60*OE and WT had no significant difference. Interestingly, the growth of lateral roots was more vigorous under −Fe, especially in *MsbHLH60OE* plants (**Figure 4A**). −Fe stress led to a decrease in root length of all plants, and the root length of *MsbHLH60*OE was significantly higher than WT (*p* < 0.05) (**Figure 4C**).

The NBT and DAB staining showed no significant difference between *MsbHLH60*OE and WT. −Fe led to a deeper staining in all plants. The leaves of the *MsbHLH60*OE were less stained than those of WT (**Figure 4D**). Under N, MDA content of *MsbHLH60*OE and WT had no significant difference. −Fe led to an increase in MDA content, with significantly lower levels in *MsbHLH60*OE than WT (*p* < 0.05) (**Figure 4E**). Under N, the GST activity of *MsbHLH60*OE was higher than WT (*p* < 0.05). Compared to N, the GST activity was significantly increased in the *MsbHLH60*OE plants by −Fe, while not in WT (*p* < 0.05) (**Figure 4F**). Under N, the contents of O_2_^-^ and H_2_O_2_ of *MsbHLH60*OE and WT had no significant difference. −Fe led to an increase in the O_2_^-^ and H_2_O_2_ contents of all plants, with significantly lower levels in *MsbHLH60*OE (p < 0.05) (**Figures 4G, H**). Under N, the activities of SOD and CAT in *MsbHLH60*OE and WT were not significantly different. −Fe led to a significant decrease in the activities of SOD and CAT of *MsbHLH60*OE and WT, with significantly higher levels in *MsbHLH60*OE (*p* < 0.05) (**Figures 4I, J**).

### 3.5 The spatiotemporal characteristics of *MsbHLH60* expression under Cd and Cd(−Fe) stress

**Figure 5:**
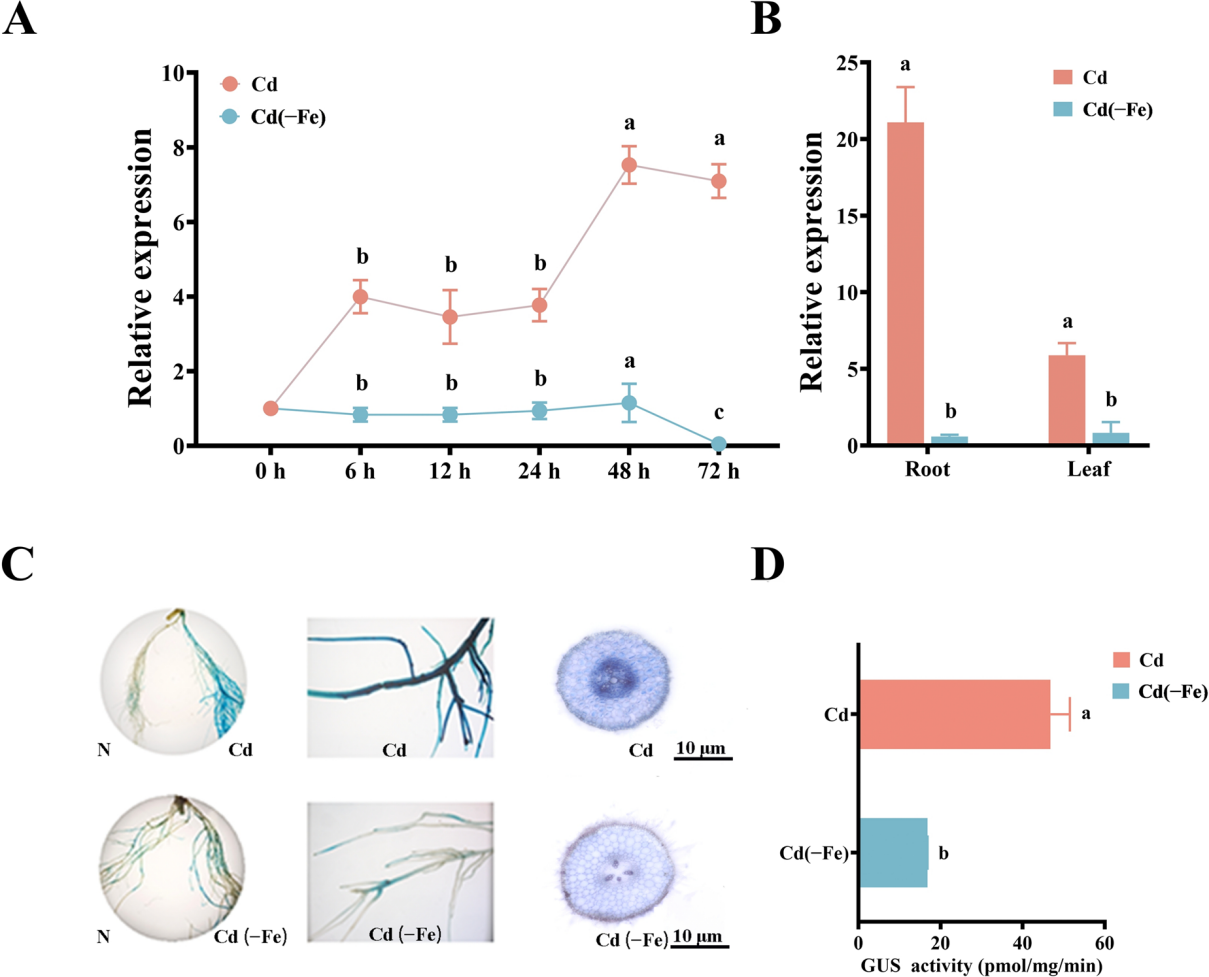
The spatiotemporal characteristics of *MsbHLH60* expression under Cd and Cd(−Fe) stress. **(A)** The relative expression of *MsbHLH60* in alfalfa seedlings under 90 µM CdCl_2_ with100 µM Fe-EDTA stress _(_Cd_)_ and 90 µM CdCl_2_ with 0 µM Fe stress (Cd(−Fe)) for 0, 6, 12, 24, 48, and 72 (h) **(B)** The relative expression of *MsbHLH60* in the root and leaf of the alfalfa under Cd, Cd(−Fe) stress for 24 (h) Statistical analysis was performed using SPSS 19.0, and graphs were generated using GraphPad Prism. A Student's t-test was used to compare transgenic plants and WT under the same treatment, and a one-way analysis of variance (ANOVA) followed by Duncan's multiple range test was used to compare the control and treatment groups. Different letters indicate a significant difference (*p* < 0.05). **(C)** GUS staining of pBI121-*MsbHLH60*pro::GUS soybean hairy root chimeras under 75 µM CdCl_2_ with 100 µM Fe-EDTA stress _(_Cd_)_ and 75 µM CdCl_2_ with 0 µM Fe stress (Cd(−Fe)) for 24 (h) From left to right: whole-root morphology, local root segment details, and transverse root section. Scale bars = 10 µm. **(D)** GUS activity measurement. Statistical analysis was performed using SPSS 19.0, and graphs were generated using GraphPad Prism. A Student's t-test was used to compare transgenic plants and WT under the same treatment, and a one-way analysis of variance (ANOVA) followed by Duncan's multiple range test was used to compare the control and treatment groups. Different letters indicate a significant difference (*p* < 0.05).

The expression of *MsbHLH60* in response to Cd and Cd(−Fe) stress was observed. Compared with untreated (0 h), the expression of *MsbHLH60* was significantly increased by Cd (*p* < 0.05) (**Figure 5A**). The expression of *MsbHLH60* continued to rise from 6 h to 48 h of Cd and then declined at 72 h. Compared with untreated (0 h), the expression of *MsbHLH60* was not affected by Cd(−Fe) from 6 h to 48 h, and significantly decreased at 72 h (*p* < 0.05) (**Figure 5A**). The expression of *MsbHLH60* in both leaf and root of alfalfa was significantly upregulated by 24 h of Cd, while downregulated by 24 h of Cd(−Fe) (*p* < 0.05) (**Figure 5B**).

Compared with N, under Cd stress, the GUS staining showed significantly deepen blue in the main roots and lateral roots of soybean hairy roots, accompanied by a significant increase in GUS enzyme activity (*p* < 0.05) (**Figures 5C, D**). The GUS staining was significantly deepened at the stele of root (**Figure 5C**). Under Cd(−Fe), the GUS staining showed shallow blue at some scattered positions of the main root, accompanied by a significantly lower GUS enzyme activity than that under Cd stress (**Figures 5C, D**).

### 3.6 Overexpression of *MsbHLH60* enhances Cd tolerance in arabidopsis

**Figure 6:**
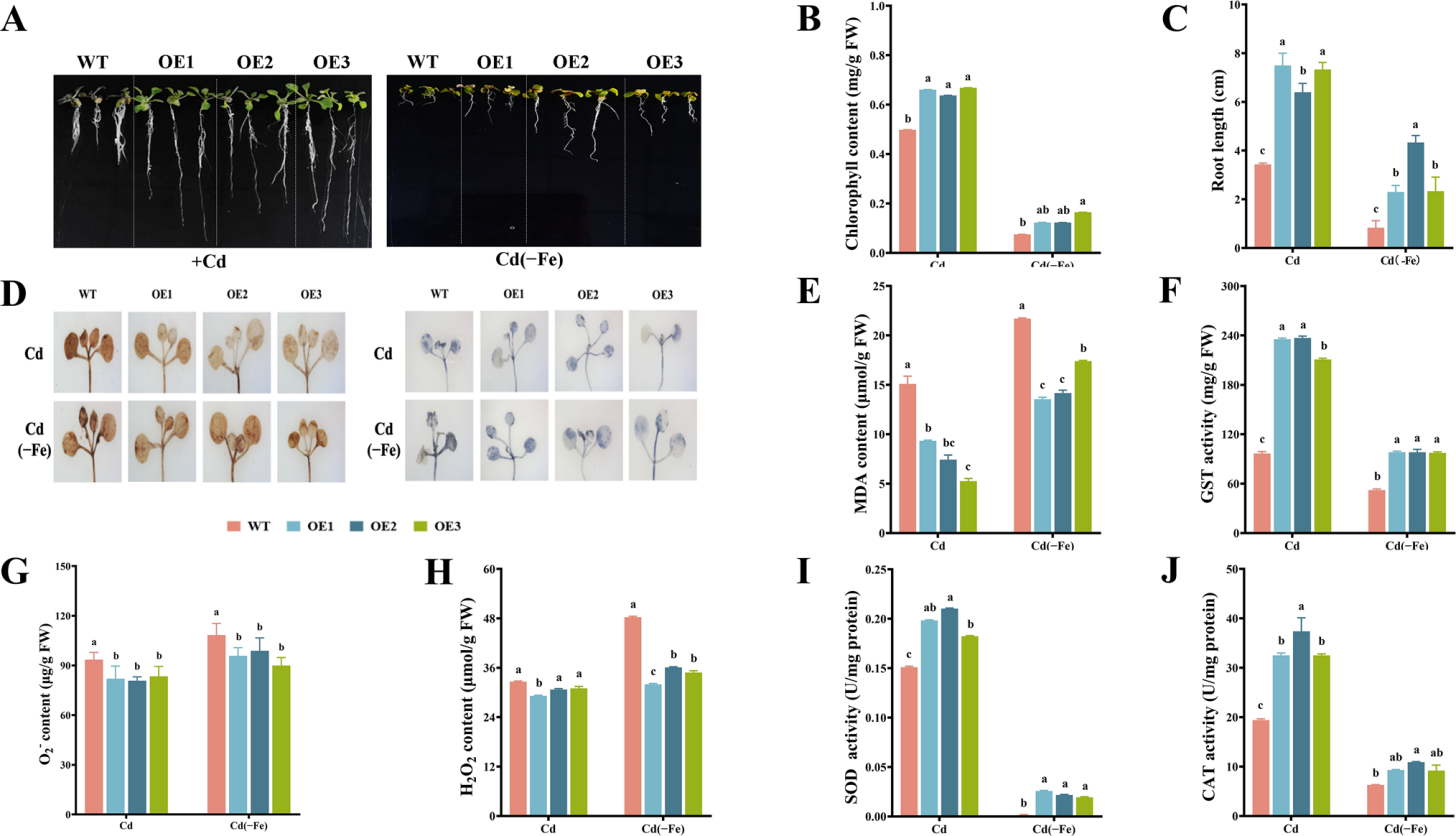
Overexpression of *MsbHLH60* enhances Cd tolerance in Arabidopsis. **(A)** Phenotypes of *MsbHLH60*OE and WT Arabidopsis seedlings under 200 µM CdCl_2_ with 100 µM Fe-EDTA stress (Cd) and 200 µM CdCl_2_ with 0 µM Fe stress (Cd(−Fe)). OE1, OE2, and OE3: T_3_ generation lines of the *MsbHLH60* transgenic Arabidopsis. **(B)** Chlorophyll content. **(C)** Root length. **(D)** DAB and NBT staining. **(E)** MDA content. **(F)** GST activity. **(G)** O_2_^−^ content. **(H)** H_2_O_2_ content. **(I)** SOD activity. **(J)** CAT activity. Statistical analysis was performed using SPSS 19.0, and graphs were generated using GraphPad Prism. A Student's t-test was used to compare transgenic plants and WT under the same treatment, and a one-way analysis of variance (ANOVA) followed by Duncan's multiple range test was used to compare the control and treatment groups. Different letters indicate a significant difference (*p* < 0.05).

Under Cd stress, *MsbHLH60*OE plants showed better growth than WT. *MsbHLH60*OE plants maintained green leaves, whereas WT leaves exhibited pronounced chlorosis (**Figure 6A**). The chlorophyll content of leaves and the root length were significantly higher in *MsbHLH60*OE than WT (*p* < 0.05) (**Figures 6B, C**). Under Cd(−Fe), the growth of both *MsbHLH60*OE and WT had been severely hindered (**Figure 6A**), although *MsbHLH60*OE still exhibited significantly higher chlorophyll content and root length (**Figures 6B, C**).

Cd(−Fe) stress led to aggravated oxidative stress indicators, specifically manifested as deep staining with NBT and DAB, lower activities of GST, SOD, and CAT, and higher levels of MDA, O_2_^−^, and H_2_O_2_ in *MsbHLH60*OE and WT (**Figures 6D, G, H**). Under Cd and Cd(−Fe), the leaves of *MsbHLH60*OE were less stained by NBT and DAB than those of WT(**Figure 6D**); Concurrently, the contents of MDA, O_2_**^−^**, and H_2_O_2_ in *MsbHLH60*OE leaves were significantly lower than those in WT(*p* < 0.05); the activities of GST, SOD, CAT in *MsbHLH60*OE leaves were significantly higher than those in WT (*p* < 0.05) (**Figures 6G, H**). In summary, overexpression of *MsbHLH60* alleviates Cd-induced oxidative damage and enhances Cd tolerance in Arabidopsis by modulating ROS accumulation, enhancing antioxidant enzyme activities, and improving detoxification capacity. Nevertheless, iron deficiency severely aggravates the injury inflicted by Cd stress and significantly diminishes this *MsbHLH60*-mediated protection.

### 3.7 MsbHLH60 assists MsFIT in regulating *MsIRT1*

**Figure 7:**
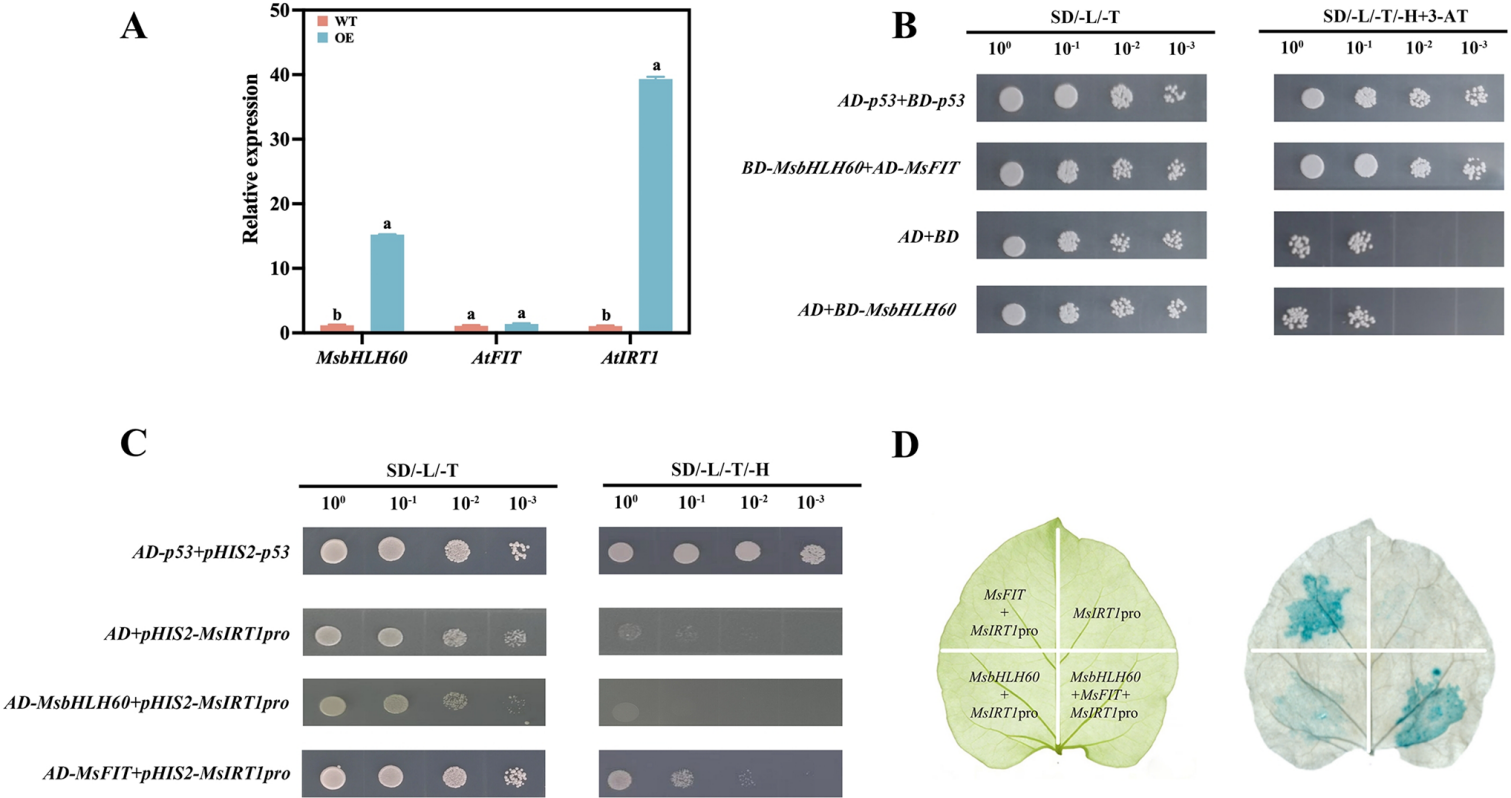
MsbHLH60 assists MsFIT in regulating *MsIRT1.* **(A)** qRT-PCR analysis of *MsbHLH60*, *AtFIT*, and *AtIRT1* expression in *MsbHLH60*-overexpressing Arabidopsis plants and WT. Statistical analysis was performed using SPSS 19.0, and graphs were generated using GraphPad Prism. A Student's t-test was used to compare transgenic plants and WT under the same treatment, and a one-way analysis of variance (ANOVA) followed by Duncan's multiple range test was used to compare the control and treatment groups. Different letters indicate a significant difference (*p* < 0.05). **(B)** Yeast two-hybrid assay of MsbHLH60 and MsFIT. The yeast cells with AD-*MsFIT* and BD-*MsbHLH60* were grown on SD/Leu/Trp and SD/Leu/Trp/His media supplemented with 3AT, indicating the interaction between MsbHLH60 and MsFIT. **(C)** Yeast one-hybrid assay of MsbHLH60 and MsFIT with the *MsIRT1* pro. Yeast cells carrying AD-*MsbHLH60* and pHIS2-*MsIRT1*pro failed to grow on both SD/-Leu/-Trp and SD/-Leu/-Trp/-His/3-AT. In contrast, cells carrying AD-*MsFIT* and pHIS2-*MsIRT1*pro grew normally, indicating a specific interaction between MsFIT and the *MsIRT1pro*, but not between MsbHLH60 and the *MsIRT1*pro. **(D)** Co-transformation driven GUS expressionin *Nicotiana benthamiana* leaves. Left: co-transformation combinations in *Nicotiana benthamiana* leaves. Right: GUS histochemical staining.

To detect the interaction between MsbHLH60 and other Iron regulation genes, the expression of *AtIRT1* and *AtFIT* in *MsbHLH60*OE Arabidopsis were examined. In addition, the interaction between MsbHLH60 and the AtFIT homologous protein MsFIT was detected *in vitro*. Moreover, the regulatory effects of MsbHLH60 and MsFIT on the *AtIRT1* homologous gene *MsIRT1* were detected *in vitro*. Compared with WT, the expression of *MsbHLH60 and AtIRT1* was significantly increased, while the expression of *AtFIT* showed no significant difference (**Figure 7A**). The pGBKT7-*MsbHLH60* recombinant vector (BD-*MsbHLH60*) was constructed (**Supplementary Figure S8**) and was used for conducting yeast two-hybridization with pGADT7-*MsFIT* (AD-*MsFIT*). Yeast two-hybrid showed that yeast could grow in the combination of BD-*MsbHLH60* with AD-*MsFIT*, indicating that MsbHLH60 can interact with MsFIT *in vitro* (**Figure 7B**). The pGADT7-*MsbHLH60* vector (AD-*MsbHLH60*) was constructed (**Supplementary Figure S9**) and was used for conducting yeast one-hybridization with pHIS2-*MsIRT1*pro. Yeast one-hybrid assay showed that yeast could not grow in the combination of AD-*MsbHLH60* and pHIS2-*MsIRT1*pro, while it could grow in the combination of AD-*MsFIT* and pHIS2-*MsIRT1*pro. These findings indicated that *MsbHLH60* did not physically interact with the *MsIRT1* promoter, whereas *MsFIT* did (**Figure 7C**).

To further determine the regulatory relationship among *MsbHLH60*, *MsFIT*, and *MsIRT1*, we conducted a transient co-transformation experiment using different gene combinations in *N. benthamiana*. Histochemical GUS staining showed that the part of the leaf transformed with *MsIRT1*pro::GUS alone displayed no obvious blue signal; the part transformed with *MsbHLH60* +*MsIRT1*pro::GUS displayed weak blue signal; the part transformed with *MsFIT* +*MsIRT1*pro::GUS displayed obvious blue signal; and the part transformed with *MsbHLH60*+*MsFIT* +*MsIRT1*pro::GUS displayed obvious and widely blue signal (**Figure 7D**). These data indicated that *MsFIT* strongly activated the transcriptional activity of the *MsIRT1*, which could be enhanced by *MsbHLH60*.

## 4 Discussion

### 4.1 The Role of *MsbHLH60* in metal long-distance transport within plant roots

Our findings indicate that *MsbHLH60* transcription levels in alfalfa roots are upregulated in response to iron deficiency, consistent with its anticipated role as a pivotal early regulator of the iron starvation response. Notably, we have directly observed that Arabidopsis plants overexpressing *MsbHLH60* exhibit robust iron stress tolerance, characterized by alterations in morphology, growth, and physiological processes. This body of evidence substantiates the role of *MsbHLH60* in detecting iron deficiency signals and modulating the expression of downstream genes. In model plants such as Arabidopsis, transcription factors belonging to the classical bHLH IVc subfamily (e.g., *FIT*) are traditionally viewed as primary regulators of genes such as *IRT1* in the root epidermis (Rodríguez-Celma et al., 2019). However, *MsbHLH60* is predominantly expressed not in the root epidermis, which is responsible for initial metal uptake, but rather exhibits significant enrichment in the cortex, pericycle, and inner regions of the central cylinder.

The findings suggest that *MsbHLH60* is involved in both lateral and radial iron transport, highlighting its previously underappreciated role in the systemic regulation of iron distribution. Notably, the transcriptional expression of *MsbHLH60* in alfalfa roots is significantly and rapidly upregulated in response to Cd exposure. Expression of *MsbHLH60*, whether induced by iron deficiency or Cd exposure, has been observed across various lateral transport tissue cell types, including cortical parenchyma, endodermis, and collenchyma sheaths (Zhao et al., 2022; Sun et al., 2025). This suggests that *MsbHLH60* regulates the transmembrane transport of iron and Cd into the collenchyma via the symplastic pathway. Furthermore, *MsbHLH60* may play a role in the selective filtration of metal ions transported through the symplastic pathway (Barberon and Geldner, 2014; Sun et al., 2025). The spatial extent of Cd-induced *MsbHLH60* expression in cortical parenchyma tissue surpasses that induced by iron deficiency. Given the broader Cd-induced expression of *MsbHLH60* in cortical parenchyma, this gene may be potentially associated with enhanced Cd compartmentation in vacuoles to relieve cytoplasmic Cd toxicity, although direct experimental evidence from subcellular fractionation and vacuolar transport assays remains to be further validated (Verbruggen et al., 2009; Ueno et al., 2010; Zhang et al., 2018).

In addition, based on its expression pattern in root transport tissues, *MsbHLH60* might be involved in modulating the activity of Cd efflux transporters, thereby redirecting Cd from the stele back to the cortex (Zhang et al., 2025a). This hypothesis is consistent with the known functions of bHLH transcription factors in regulating Cd efflux transporters, such as *bHLH104* in Arabidopsis (Yao et al., 2018). It is also supported by studies on vascular-specific regulation of Cd transporters, as exemplified by *BrpHMA2* in Brassica parachinensis (Liu et al., 2022a). Furthermore, bHLH transcription factors have been increasingly recognized as key components of the regulatory network underlying Cd stress responses (Li et al., 2023a). Following absorption, Fe and Cd ions must be loaded and unloaded into xylem vessels within the central column to enable their upward transport via transpiration to the aerial parts (Doblas et al., 2017). Plants overexpressing *MsbHLH60* under iron-deficient conditions exhibit pronounced chlorosis, whereas under iron-sufficient conditions, the overexpression of *MsbHLH6*0 ameliorates chlorosis even in the presence of Cd. This, combined with *MsbHLH60* expression in the primary xylem, suggests its role in the radial transport of iron and Cd from roots to shoots, implying that it may directly regulate key genes involved in xylem loading. *MsbHLH60* may also play a role in the storage of metal ions within xylem parenchyma cells and in the lateral redistribution of ions within and outside the xylem (Plavcová and Jansen, 2015). The findings suggest that *MsbHLH60* plays a role in Cd retention within the primary xylem, thereby limiting its translocation to aerial plant parts (Pan et al., 2024). The expression profile of *MsbHLH60* in roots suggests its involvement in metal stress sensing and signal transduction, as well as in the regulation of metal unloading and loading during root transport. This may constitute a regulatory mechanism by which alfalfa adapts to its perennial growth habit, extensive root system, and substantial nutrient allocation demands (Huang et al., 2024).

### 4.2 MsbHLH60 interacts with MsFIT to co-activate *MsIRT1* expression

Our experimental data indicate that heterologous expression of *MsbHLH60* in Arabidopsis activates the expression of *AtIRT1*, the key iron transport gene in Arabidopsis. This finding demonstrates that this core regulatory module is highly conserved between alfalfa and other dicotyledonous plants (Rodríguez-Celma et al., 2019; Zhang et al., 2023). Although MsbHLH60 does not directly bind to the promoter of *MsIRT1*, its overexpression in Arabidopsis significantly upregulates *AtIRT1* transcript levels. Moreover, MsbHLH60 has no regulatory effect on the expression of *AtFIT*, but physically interacts with MsFIT.

The influence of MsbHLH60 on *AtIRT1* expression in *MsbHLH60*OE Arabidopsis may be attributed to indirect regulatory mechanisms, similar to members of the bHLH Ib subfamily, such as AtbHLH38/39/100/101, form heterodimers with AtsFIT to augment its activation of *AtIRT1* expression (Yuan et al., 2008; Wang et al., 2013). We propose that in alfalfa, MsbHLH60 confers tissue specificity and promotes the recognition of downstream target gene promoters by MsFIT, and together with MsFIT forms a functionally complete transcriptional activation complex whose activity is finely modulated by iron nutritional status and tissue-specific traits. This pathway allows for the precise regulation of tissue iron distribution by concurrently managing iron-deficiency-induced FIT accumulation (“signal”) and tissue-specific recognition (“tissue sensor,” bHLH60). This regulatory pattern likely represents an optimization of the iron-uptake regulatory network in perennial legumes adapted to particular ecological niches, such as calcareous soils. The enhancement of regulation at a core node *MsFIT* via *MsbHLH60* enables more rapid and precise responses to fluctuations in iron availability. This strongly underscores the significance of the *MsbHLH60* in alfalfa and potentially in other related species.

### 4.3 *MsbHLH60* functions as a critical integrative node within the regulatory networks of iron deficiency and Cd response

It is well established that transporters such as *IRT1* possess relatively broad substrate specificity, rendering them unable to effectively distinguish between chemically similar ions, such as Fe^2+^ and Cd^2+^. As a result, signals that prompt increased iron uptake during iron deficiency also inadvertently enhance Cd absorption (Spielmann et al., 2022; Cointry et al., 2025). In environments characterized by iron deficiency and Cd, plants often face the dual challenge of addressing “iron starvation” while risking “Cd toxicity” (McInturf et al., 2022). The spatiotemporal expression pattern of *MsbHLH60* reveals its induction by iron-deficiency signals to regulate long-distance iron transport, thereby prioritizing the allocation of limited iron resources to organs with the greatest need. Empirical evidence indicates that *MsbHLH60* increases chlorophyll content in plants subjected to Cd stress (under normal iron conditions), suggesting that it enhances the upward transport of iron in the presence of Cd, thus providing essential raw materials for chlorophyll synthesis.

The upregulation of *MsbHLH60* in the mesophyll, coupled with increased GST enzyme activity under Cd stress, strongly suggests that *MsbHLH60* may modulate downstream targets. This modulation could either restrict Cd influx into sensitive regions or facilitate its sequestration in non-essential organs, such as roots (Jiang et al., 2024). These represent two distinct regulatory mechanisms: one targeting transporters that facilitate metal movement and the other involving chelating complexes that immobilize metals. When considered independently, the role of *MsbHLH60* in both the response to iron deficiency and Cd stress is undoubtedly beneficial to plants, as it alleviates nutrient limitations and mitigates metal toxicity. However, when Cd and iron deficiency signals are coupled, the iron transport genes co-activated by *MsbHLH60* may result in significant Cd influx into the plant’s aerial parts. Conversely, Cd-activated metal immobilization genes may hinder the redistribution of limited iron within the plant (Ivanov et al., 2012). In this context, the reduced expression of *MsbHLH60* in most root tissues likely reflects a plant’s trade-off between “nutrient acquisition” and “toxicity avoidance”, representing a survival strategy. This suggests that *MsbHLH60* functions as a pivotal regulatory element in this balance.

### 4.4 Functional model of *MsbHLH60* in iron deficiency and Cd stress response regulatory networks

The beneficial effect of *MsbHLH60* overexpression in mitigating the adverse impacts of Cd stress and iron deficiency stress is clearly demonstrated. The findings indicate that these two regulatory networks may share components of signal transduction and response mechanisms (McInturf et al., 2022). Within the antioxidant defense system, overexpression of *MsbHLH60* effectively reduced ROS levels induced by both Cd and iron deficiency stresses, resulting in higher antioxidant enzyme activity than in WT plants. Under conditions of iron deficiency and Cd stress, the increase in H_2_O_2_ levels and the decrease in SOD activity were most significant, yet *MsbHLH60* overexpression still conferred a protective effect. Notably, it was observed that a deficiency in iron or Cd triggers the expression of *MsbHLH60* in the pericycle. The observed enhancement in primary root elongation and lateral root development in transgenic Arabidopsis under stress conditions suggests that *MsbHLH60* may have a role beyond the direct regulation of iron uptake proteins, extending to the modulation of root morphogenesis in response to iron homeostasis.

*MsbHLH60* is posited to regulate lateral root formation, thereby modifying root architecture to adapt to metal-rich environments and increasing root-surface contact with the soil, which may indirectly influence root iron uptake (Zhang et al., 2022; Yalamanchili et al., 2024). Furthermore, *MsbHLH60* has been observed to mitigate Cd-induced inhibition of root growth, suggesting its potential role in promoting root development as a growth-based response to metal toxicity. Results from treatments involving Cd and iron deficiency (Cd(−Fe)) indicate that iron deficiency directly leads to the collapse of the photosynthetic apparatus and a reduction in energy metabolism. When combined with Cd-induced damage, such as the production of reactive oxygen species and enzyme inactivation, plants approach mortality, at which point the mitigating effects of *MsbHLH60* overexpression become limited. This strongly suggests that iron is a critical prerequisite for *MsbHLH60*’s ability to confer tolerance to Cd stress, thereby balancing the iron-Cd trade-off. Based on these findings, we propose a working model in which *MsbHLH60* functions as an integrative hub coordinating responses to iron nutrition and Cd stress. Upon detection of iron deficiency signals by the roots, upstream systemic signals (e.g., hormones, peptides) or local rhizosphere redox signals activate *MsbHLH60*.

Future studies will precisely map *MsbHLH60*’s genome-wide binding sites under various stress conditions to directly validate the reprogramming of its downstream target gene profiles. Furthermore, we will generate *MsbHLH60* knockout mutants to confirm its essentiality under diverse treatment conditions.

## 5 Conclusion

In summary, this study demonstrated that the alfalfa transcription factor *MsbHLH60*, a bHLH protein localized in both the nucleus and cytoplasm, exhibits differential responses to iron deficiency and Cd stress. Heterologous overexpression of *MsbHLH60* enhances the tolerance of Arabidopsis to iron deficiency and Cd toxicity, whereas the Cd tolerance of Arabidopsis is attenuated by iron deficiency. In *MsbHLH60*OE Arabidopsis, *MsbHLH60* significantly induces the transcriptional expression of *AtIRT1* but does not activate the expression of *AtFIT*, the upstream regulatory gene of *AtIRT1*. In the interaction verification experiments *in vitro*, MsbHLH60 physically interacts with MsFIT, while not binding to the *MsIRT1* promoter. The results of this research support that *MsbHLH60* could enhance the transcriptional activation effect of *MsFIT* on *MsIRT1*, thereby playing an auxiliary regulatory role in regulating iron homeostasis and Cd stress adaptability in plants.

## Data Availability

The original contributions presented in the study are included in the article/**Supplementary Material**. Further inquiries can be directed to the corresponding author.
