# Supplementary material for: Iron status modulates cadmium tolerance: mechanistic insights from MsbHLH60-mediated regulation of the FIT/IRT1 module in alfalfa
Source: Front Plant Sci. 2026 May 8;17:1821873. doi: 10.3389/fpls.2026.1821873 (PMC13193905; doi:10.3389/fpls.2026.1821873)
Supplement: Supplementary file 1 [file SupplementaryFile1.docx]

Supplementary Material

# Supplementary Table S1. Primer sequences used in this study.

| **Primer name** | **Primer sequence（5ʹ-3ʹ）** | **Purpose** |
| --- | --- | --- |
| *MsbHLH60*-S | GCTCTAGAATGGGATCAGAGAGTGCG | Cloning for pBI121-*MsbHLH60* |
| *MsbHLH60*-A | CCCCCGGGTCAACTTGAAGTTGGTTGCT |  |
| *MsbHLH60*(Y)-S | CTCTAGAATGGGATCAGAGAGTGCG | Cloning for pGADT7-*MsbHLH60*、pGBKT7-*MsbHLH60* |
| *MsbHLH60*(Y)-A | CCCCCGGGTCAACTTGAAGTTGGTTGCT |  |
| *MsbHLH60*-GFP-S | GCTCTAGAATGGGATCAGAGAGTGCG | Cloning for pBI121-*MsbHLH60*-GFP |
| *MsbHLH60*-GFP-A | CCCCCGGGACTTGAAGTTGGTTGCTTTC |  |
| *MsbHLH60*(Q)-S | GGTGTTGTTCAAAGCTCTCCAG | qRT-PCR |
| *MsbHLH60*(Q)-A | TCATACCAATCTTATCTTCCTCCC |  |
| *MsActin11*-S | GCGGGAAATTGTAAGGGATGT |  |
| *MsActin11*-A | TCGCCAATAGTGATGACCTG |  |
| *AtFIT(*Q)-S | ACCCGCTGTTCCTGATAC |  |
| *AtFIT*(Q)-A | CCGAACCCCATACTGTTGTAAT |  |
| *AtIRT1*(Q)-S | AACCCTCCTTCCTCCCATATG |  |
| *AtIRT1*(Q)-A | TCGCACTTTCTCACGACCTG |  |
| *AtActin2*(Q)-S | TAAGGGTGGTGCCAAGAAGGT |  |
| *AtActin2*(Q)-A | AGCAAGAGGAGCAAGGCACTT |  |
| *MsbHLH60*pro-S | AATGGAAGATGAGGAGGAT | Cloning for pBI121-*MsbHLH60*pro::GUS |
| *MsbHLH60*pro-A | GTGATTAAAGCCAGTTATATG |  |
| *MsIRT1*pro-S | CGGAATTCCCACCCCACGAGACTAA | Cloning for pHIS2-*MsIRT1*pro |
| *MsIRT1*pro-A | CGAGCTCTGTATAGTTACTGAATAAGCCAT |  |

# Supplementary Table S2. The physicochemical properties of *MsbHLH60*

| **Gene Name** | **Number of Amino Acids** | **Molecular Weight** | **Theoretical pI** |
| --- | --- | --- | --- |
| *MsbHLH60* | 321 | 35267.25 | 5.72 |

**Supplementary Table S3. Interspecific Syntenic Homologous Gene Correspondence Table between *Medicago sativa* and *Medicago truncatula***

| ***M.truncatula* Chromosome No.** | ***M. truncatula* Gene ID** | **Syntenic Relationship** | ***M.sativa***  **Chromosome No.** | ***M. sativa* Gene ID** |
| --- | --- | --- | --- | --- |
| chr2 | Medtr2g101520.1 | == | chr2_1 | Msa0225110 |
| chr2 | Medtr2g101520.1 | == | chr2_2 | Msa0266200 |
| chr6 | Medtr6g488100.2 | == | chr4_1 | Msa0574080 |
| chr6 | Medtr6g488100.2 | == | chr6_3 | Msa0965520 |
| chr6 | Medtr6g488100.2 | == | chr6_4 | Msa1000020 |
| chr8 | Medtr8g103065.1 | == | chr4_1 | Msa0574080 |
| chr8 | Medtr8g103065.1 | == | chr6_3 | Msa0965520 |

**Supplementary Table S4. Table of SUMO Modification Analysis of *MsbHLH60* Protein.**

| **ID** | **Position** | **Peptide** | **Score** | **Cut-off** | **Type** | **Source** | **PPI** |
| --- | --- | --- | --- | --- | --- | --- | --- |
| *MsbHLH60* | 96 | NASLVDVKQENSGNS | 0.9588 | 0.67 | SUMOylation | Pred. | PPI |
| *MsbHLH60* | 92-96 | SQAPNASLVDVKQENSGNS | 0.9477 | 0.5 | SUMO interaction | Pred. | PPI |
| *MsbHLH60* | 278-282 | KDLKSRGLCLVPVSCTLQV | 0.8371 | 0.5 | SUMO interaction | Pred. | PPI |
| *MsbHLH60* | 171 | STTQTTFKVRKEKLG | 0.8194 | 0.67 | SUMOylation | Pred. | PPI |
| *MsbHLH60* | 157 | SASGGAMKKARVQQS | 0.8173 | 0.67 | SUMOylation | Pred. | PPI |
| *MsbHLH60* | 270 | EVSQEEPKKDLKSRG | 0.8028 | 0.67 | SUMOylation | Pred. | PPI |
| *MsbHLH60* | 274 | EEPKKDLKSRGLCLV | 0.7893 | 0.67 | SUMOylation | Pred. | PPI |
| *MsbHLH60* | 199-203 | GKTDTASVLLEAIGYIRFL | 0.7536 | 0.5 | SUMO interaction | Pred. | PPI |
| *MsbHLH60* | 72 | GIVGEEDKIGMSQFQ | 0.7233 | 0.67 | SUMOylation | Pred. | PPI |


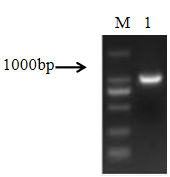


**Supplementary Figure S1. PCR amplification of the *MsbHLH60* gene**

M: DL2000; 1: *MsbHLH60* gene


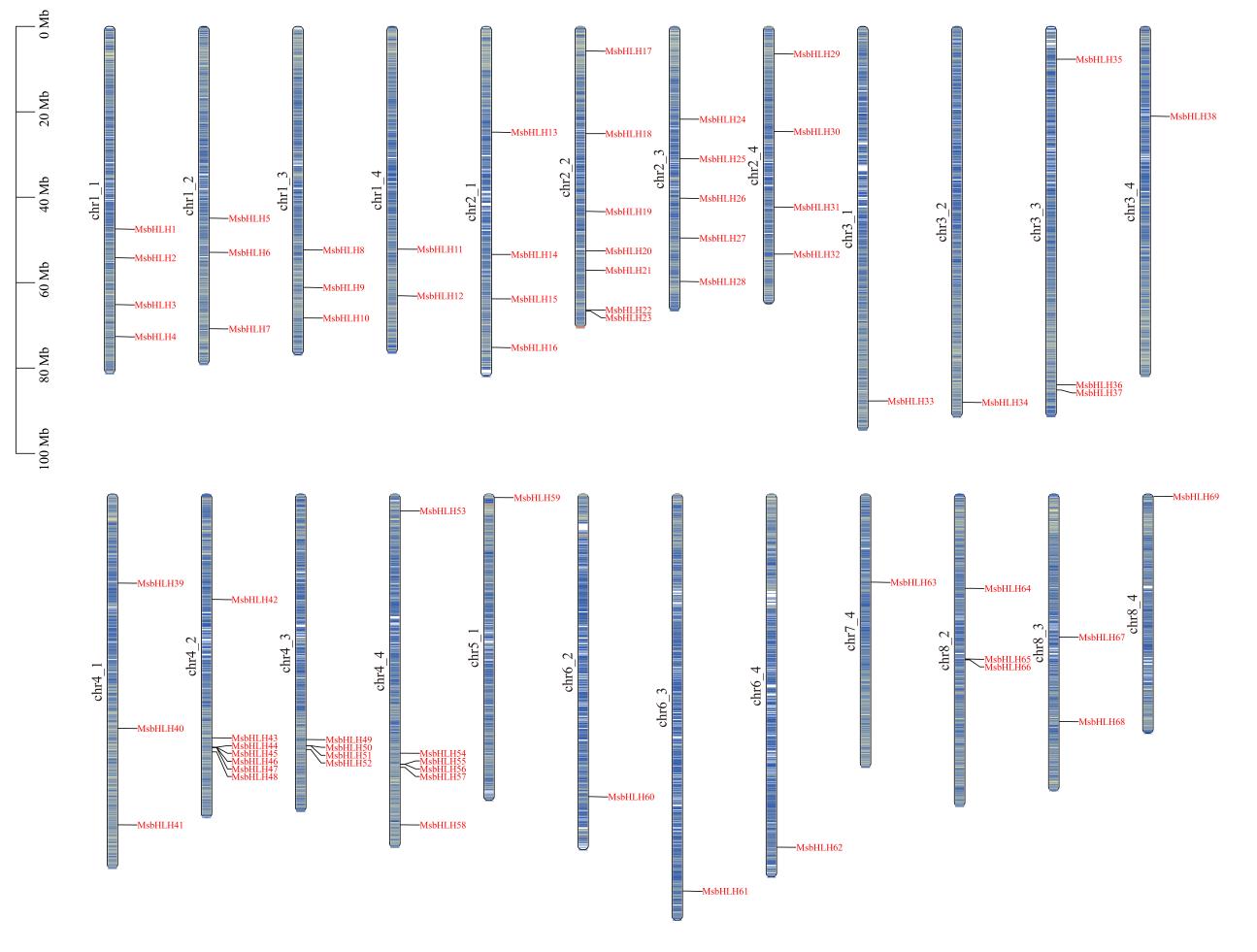


**Supplementary Figure S2. Chromosomal localization of MsbHLH family members (containing the bHLH_AtbHLH-like domain) in *Medicago sativa***

Chromosomal distribution of *M. sativa* MsbHLH genes encoding proteins with the bHLH_AtbHLH_like domain: Genes are mapped to *M. sativa* chromosomes (chr1–chr8; physical positions indicated in megabases, Mb). The gene localized to the corresponding chromosomal region (red label) is designated MsbHLH60, with its nomenclature assigned based on this genomic position (following the annotation framework for MsbHLH genes containing the bHLH_AtbHLH_like domain in *M. sativa*).


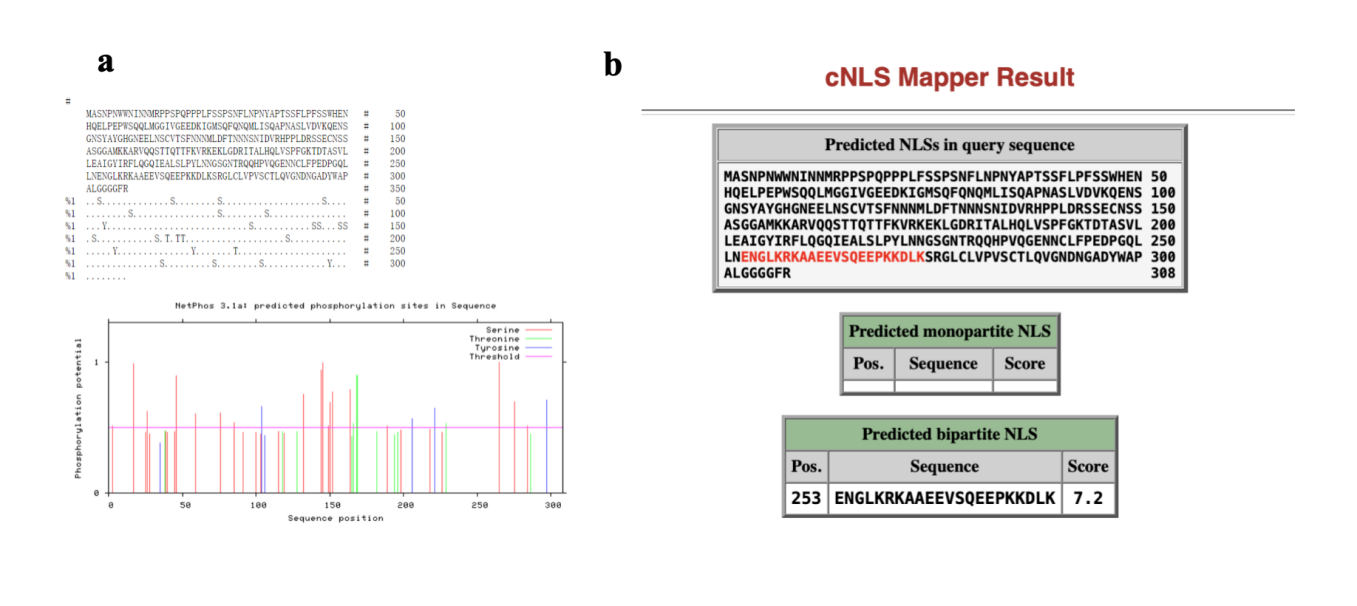


**Supplementary Figure S3. Phosphorylation site prediction and nuclear localization signal (NLS) analysis of MsbHLH60**

(a) Phosphorylation site prediction of MsbHLH60 via NetPhos 3.1: The plot shows predicted phosphorylation potential (y-axis) across the amino acid sequence (x-axis), with distinct signals for serine (red), threonine (green), and tyrosine (blue) residues. Multiple high-potential sites (e.g., serine residues) are distributed throughout the sequence. (b) NLS prediction of MsbHLH60 via cNLS Mapper: A bipartite NLS (positions 253–274; sequence: ENGLK RKAAEEVSQEEPKKDLK) was predicted with a score of 7.2, confirming the nuclear localization potential of MsbHLH60.


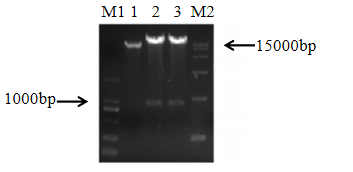


**Supplementary Figure S4. The identification by double digestion of pBI121-*MsbHLH60*-GFP**

M1: DL2000; 1: Recombinant plasmids pBI121-*MsbHLH60*-GFP; 2-3: Digestion products of pBI121-*MsbHLH60*-GFP by *Xba* I and *Sma* I; M2: DL15000.


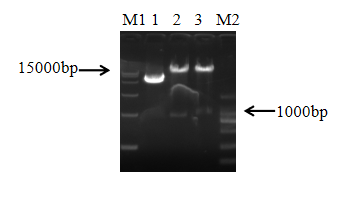


**Supplementary Figure S5. The identification by double digestion of pBI121-*MsbHLH60*pro::GUS**

M1: DL15000; 1: Recombinant plasmids pBI121-*MsbHLH60*pro::GUS ; 2-3: Digestion products of pBI121-*MsbHLH60*pro::GUS by *Bgl* II and *Xba* I ; M2: DL2000.


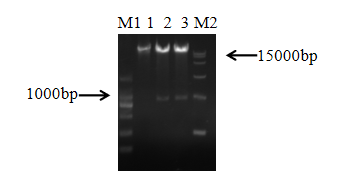


**Supplementary Figure S6. The identification by double digestion of pBI121-*MsbHLH60***

M1: DL2000; 1: Recombinant plasmids pBI121-*MsbHLH60*; 2-3: Digestion products of pBI121-*MsbHLH60* by *XbaI、SmaI* ; M2: DL15000.


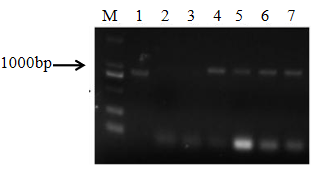


**Supplementary Figure S7. PCR identification of *MsbHLH60-*overexpressing *Arabidopsis thaliana***

M: DL2000;1: Positive control; 2: Negative control; 3: H_2_O; 4-7: PCR products


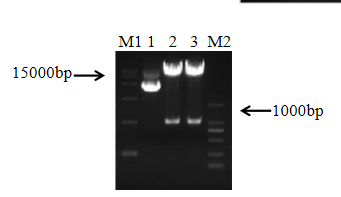


**Supplementary Figure S8. The identification by double digestion of pGBKT7-*MsbHLH60***

M1: DL15000; 1: Recombinant plasmids pGBKT7-*MsbHLH60*; 2-3: Digestion products of pGBKT7-*MsbHLH60* by *Xba* I and *BamH* I ; M2: DL2000.


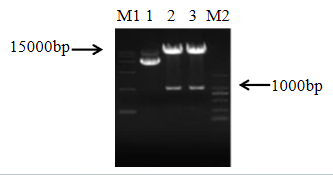


**Supplementary Figure S9. The Identification by double digestion of pGADT7-*MsbHLH60***

M1: DL15000; 1: Recombinant plasmids pGADT7-*MsbHLH60*; 2-3: Digestion products of pGADT7-*MsbHLH60* by *Xba* I and *BamH* I ; M2: DL2000.
